# Immunoglobulin G4-related disease: autoimmune pancreatitis and extrapancreatic manifestations*

**DOI:** 10.1590/0100-3984.2013.1911

**Published:** 2016

**Authors:** Daniel Alvarenga Fernandes, Ricardo Yoshio Zanetti Kido, Ricardo Hoelz de Oliveira Barros, Daniel Lahan Martins, Thiago José Penachim, Nelson Marcio Gomes Caserta

**Affiliations:** 1 MD, Attending Radiologist in the Radiology Department of the Faculdade de Ciências Médicas da Universidade Estadual de Campinas (FCM-Unicamp), Campinas, SP, Brazil.; 2 MD, Resident in Radiology and Diagnostic Imaging at the Faculdade de Ciências Médicas da Universidade Estadual de Campinas (FCM-Unicamp), Campinas, SP, Brazil.; 3 MD, MSc, Attending Radiologist in the Radiology Department of the Faculdade de Ciências Médicas da Universidade Estadual de Campinas (FCM-Unicamp), Campinas, SP, Brazil.; 4 MD, Attending Radiologist in the Radiology Department of the Faculdade de Ciências Médicas da Universidade Estadual de Campinas (FCM-Unicamp), at the Pontifícia Universidade Católica de Campinas (PUC-Campinas), and at the Centro Radiológico Campinas - Hospital Vera Cruz, Campinas, SP, Brazil.; 5 PhD, Tenured Professor in the Radiology Department of the Faculdade de Ciências Médicas da Universidade Estadual de Campinas (FCM-Unicamp), Campinas, SP, Brazil.

**Keywords:** Pancreatitis, Autoimmune diseases, Pancreas, Computed tomography, Magnetic resonance imaging

## Abstract

We present a case of immunoglobulin G4 (IgG4)-related disease with pancreatic and extrapancreatic involvement, including the biliary and renal systems. Given the importance of imaging methods for the diagnosis of IgG4-related disease and its differentiation from pancreatic adenocarcinoma, we emphasize important abdominal computed tomography and magnetic resonance imaging findings related to this recently recognized systemic autoimmune disease.

## Abstract (pt)

Apresentamos um caso de doença relacionada à IgG4, com acometimento pancreático e extrapancreático biliar e renal. Dada a relevância dos métodos de imagem para seu diagnóstico e diferenciação com o adenocarcinoma pancreático, ressaltamos os principais achados abdominais na tomografia computadorizada e ressonância magnética desta doença autoimune sistêmica recentemente reconhecida.

## INTRODUCTION

Immunoglobulin G4 (IgG4)-related disease is a systemic autoimmune disease with pancreatic and extrapancreatic abdominal manifestations; it can affect the bile ducts, kidneys, lymph nodes, prostate, and retroperitoneum, occurring primarily in the 6th and 7th decades of life^(1,2)^. Although the full range of visceral involvement has yet to be fully described, involvement of the salivary glands, periorbital tissues, meninges, lungs, aorta, pericardium, breasts, thyroid, and skin have been reported. Given that it most commonly affects the pancreas, IgG4-related disease commonly presents clinically as abdominal discomfort, obstructive jaundice, and weight loss, the differential diagnosis with pancreatic adenocarcinoma being important^(3,4)^.

The diagnosis of IgG4-related disease is based on clinical, radiological, and pathological findings. Given the importance of imaging methods for the diagnosis of IgG4-related disease, we report a case of IgG4-related disease, highlighting the main computed tomography (CT) and magnetic resonance imaging (MRI) findings.

## CASE REPORT

A 49-year-old male patient presented with pruritus, abdominal discomfort, jaundice, choluria, hypocholic stools, and weight loss (of 13 kg in five months). Physical examination revealed jaundice and no fever, as well as a soft abdomen that was painful on palpation of the right side. The patient had a history of smoking and alcoholism. Laboratory tests showed elevated liver enzymes and bilirubin levels (with an obstructive pattern), serum amylase and lipase levels being within the normal range.

After an ultrasound showing bile duct dilatation, an endoscopic retrograde cholangiopancreatography revealed common bile duct stenosis. An endoscopic papillotomy was performed, and a biliary stent was placed. Brush biopsy of the bile duct was negative for neoplasia.

**Figure 1:**
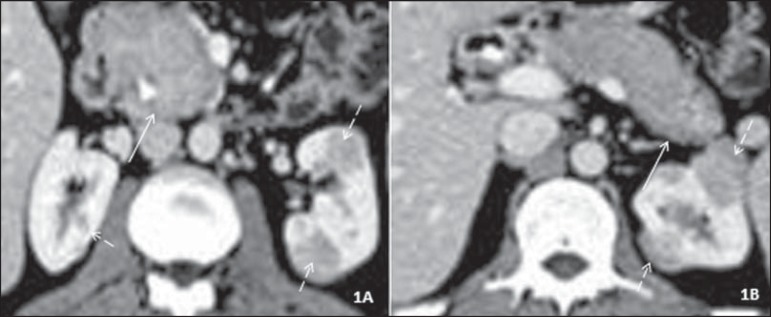
Axial contrast-enhanced CT scan of the abdomen, nephrographic phase. **A:** diffuse pancreatic enlargement (solid arrow). Note also a biliary stent in the common bile duct and hypoenhancing cortical nodules in both kidneys (dashed arrows). **B:** Retraction and obliteration of the pancreatic tail, together with a hypoenhancing halo (solid arrow), in addition to the renal nodules.

**Figure 2:**
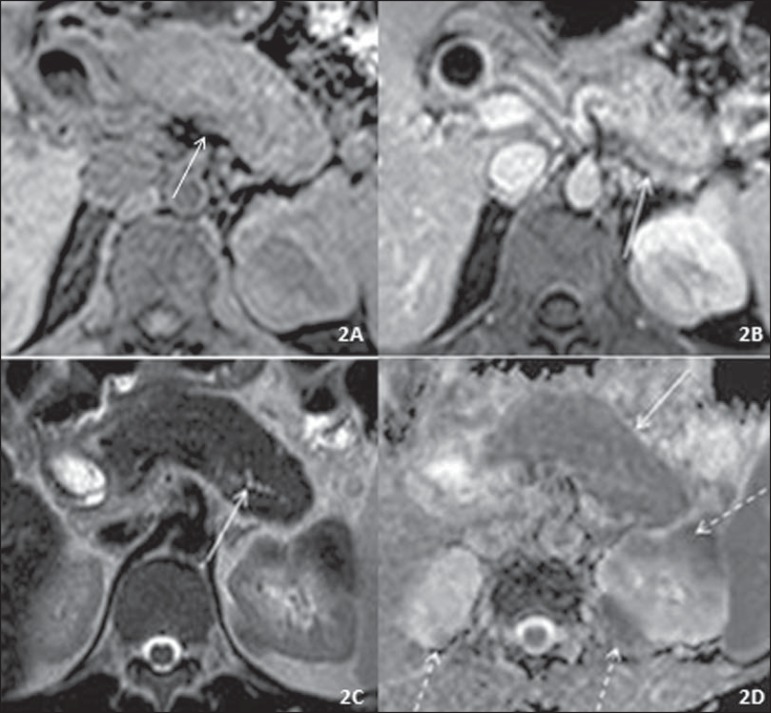
Axial abdominal MRI scan. **A:** T1-weighted image without contrast. Diffusely increased, slightly hypointense pancreas with loss of the normal pancreatic roughness (solid arrow). Note also a hypointense renal nodule. **B:** Contrast-enhanced T1-weighted image, portal phase. Hypoenhancing peripancreatic halo (solid arrow), together with hypoenhancing, partially ill-defined renal nodules. **C:** T2-weighted image. Diffusely increased pancreas, shortened pancreatic tail, and loss of the normal pancreatic roughness (a “sausage-like” pancreas), as well as diffuse, irregular narrowing of the main pancreatic duct (solid arrow), together with hypoenhancing, partially ill-defined renal nodules. **D:** Diffusion restriction in the entire pancreas, with an ADC value of 0.839 × 10–3 mm^2^/s (solid arrow), as well as in the renal nodules (dashed arrows).

**Figure 3:**
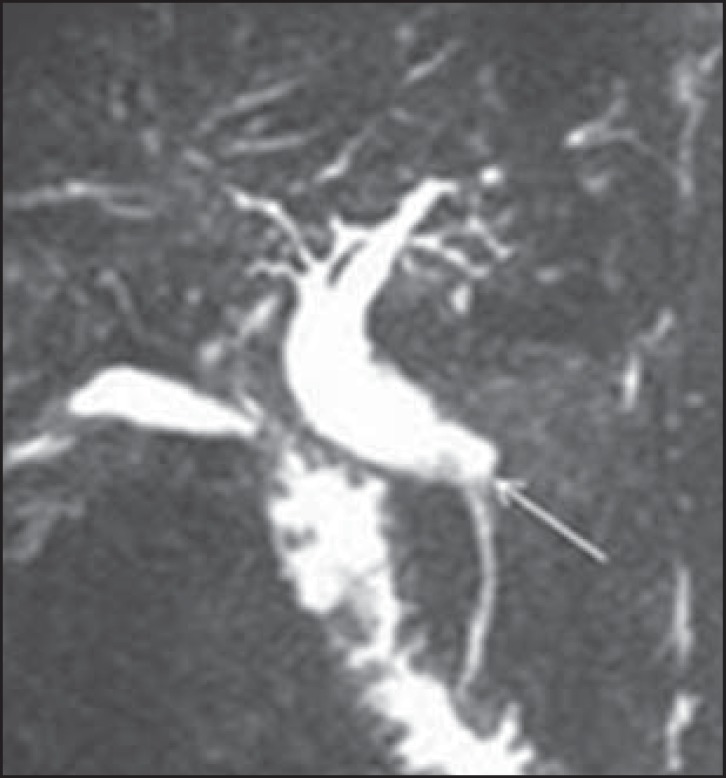
Magnetic resonance cholangiography showing bile duct dilatation up to the intrapancreatic common bile duct (arrow).

Abdominal CT and MRI scans showed diffuse pancreatic enlargement with loss of the normal pancreatic lobulation and roughness, a hypoenhancing peripancreatic halo in the tail of the pancreas, and hypoenhancing renal nodules with partially ill-defined borders (Figures 1 and 2). Magnetic resonance cholangiography showed bile duct dilatation (Figure 3).

**Figure 4:**
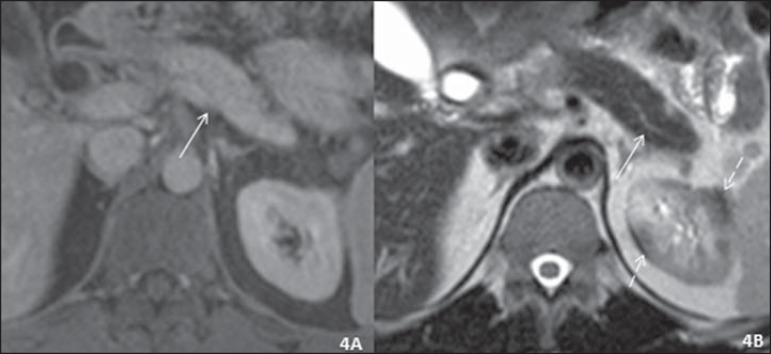
Axial abdominal MRI scan taken after corticosteroid therapy. **A:** Contrast-enhanced T1-weighted image, portal phase. Normally sized pancreas without the peripancreatic halo seen before (solid arrow). No renal nodules were seen on T1-weighted images. **B:** T2-weighted image. Normal-sized pancreas without the main pancreatic duct changes seen previously (solid arrow). Small nodules remain, seen only on T2-weighted images (dashed arrows) and smaller in size than those observed before corticosteroid therapy.

Given the suspicion of IgG4-related disease, renal and pancreatic biopsies were performed. Examination of the biopsy samples showed dense, mixed lymphoplasmacytic inflammatory infiltrate with no evidence of epithelial neoplasia, most of the plasma cells in the infiltrate being IgG4-positive. Taken together, the imaging and histopathological findings confirmed the diagnosis of IgG4-related disease; the patient was started on corticosteroid therapy, which resulted in significant clinical and radiological improvement after approximately 16 weeks (Figure 4). At this writing, the patient is receiving outpatient follow-up care and responding well to treatment.

## DISCUSSION

IgG4-related disease is a recently recognized disease that was described as a specific clinical and pathological entity in 2003^(1)^. It is characterized by lesions showing fibrosis-associated inflammatory response and lymphoplasmacytic infiltration rich in tissue IgG4-positive plasma cells, constituting a spectrum of fibroproliferative diseases including autoimmune pancreatitis, sclerosing cholangitis, sclerosing sialadenitis, sclerosing mesenteritis, inflammatory pseudo-tumor, and retroperitoneal fibrosis.

According to the international consensus diagnostic criteria for autoimmune pancreatitis, there are two specific subtypes of autoimmune pancreatitis (types 1 and 2), which can be differentiated on the basis of five cardinal criteria: a) imaging changes in the pancreatic parenchyma and duct; b) serology (for IgG4 and IgG antinuclear antibodies); c) extra-pancreatic involvement; d) histology; e) response to corticosteroid therapy^(5)^.

The diagnosis of type 1 and 2 autoimmune pancreatitis can be definitive or probable, and, in many cases, the distinction between the two subtypes is impossible. Type 1 autoimmune pancreatitis is also known as lymphoplasmacytic sclerosing pancreatitis and is the most common manifestation of IgG4-related disease, pancreatic involvement being classified as diffuse, focal, or multifocal. In the case presented here, a probable diagnosis of type 1 autoimmune pancreatitis was established. Diffuse pancreatic involvement is the most common type of pancreatic involvement and includes diffuse pancreatic enlargement with loss of the normal pancreatic lobulation and roughness (a "sausage-like" pancreas), which is due to the lymphoplasmacytic infiltrate and obliterative phlebitis associated with IgG4-related disease. On MRI scans, the pancreas appears slightly hyperintense on T2-weighted images and hypointense on T1-weighted images; a thin, hypointense halo is sometimes seen on T1- and T2-weighted images. In 16-80% of cases, there is delayed, mild homogeneous enhancement after contrast administration, with a thin, hypoenhancing peripancreatic halo, indicating inflammatory and fibrotic changes in peripancreatic tissues, as well as irregular narrowing of the main pancreatic duct^(2-4)^, all of which were observed in the case presented here. In cases of focal or multifocal involvement, there are relatively well-defined lesions associated with ductal stenosis and upstream dilatation^(1)^.

Autoimmune pancreatitis can mimic pancreatic adenocarcinoma, especially when the disease is limited to the head of the pancreas (as it is in approximately 80% of cases). The two entities can be differentiated by determining the following: whether or not the main duct penetrates the mass-it does in cases of pancreatitis, but it does not in cases of neoplasia^(2-4)^; the intensity of contrast enhancement, which is lower in cases of adenocarcinoma than in cases of pancreatitis^(3)^; and apparent diffusion coefficient (ADC) values, which are lower in cases of pancreatitis than in cases of adenocarcinoma, a cut-off ADC value of 1.075 × 10^-3^ mm ^2^/s having been suggested for differentiating between the two entities^(6)^. Cases of diffuse pancreatic involvement should be differentiated, first and foremost, from acute pancreatitis; in cases of IgG4-related disease, there is a peripancreatic halo, little or no peripancreatic densification, and no fat necrosis. In most cases, however, a combination of clinical, laboratory, imaging, and histological criteria is required for such differentiation^(2-4)^.

With regard to extrapancreatic manifestations, the biliary tree is the most common site of involvement; bile duct wall thickening, stenosis, irregularity, and upstream dilatation can occur, the intrapancreatic portion being the most commonly affected site^(1-4)^. These findings are similar to those in patients with primary sclerosing cholangitis (PSC), the differential diagnosis between the two entities posing a challenge. Patients with PSC are generally less symptomatic, whereas those with IgG4-related sclerosing cholangitis have disease that is more acute and of a shorter duration. Patients with PSC have multifocal involvement of short segments including intrahepatic or extrahepatic bile ducts-normal segments alternating with slightly dilated segments to produce a "beaded" appearance-whereas patients with IgG4-related sclerosing cholangitis have strictures that typically affect a long segment and are continuous with prestenotic dilatation^(2-4)^. Given this challenge, the American Association for the Study of Liver Diseases recommends that serum IgG4 levels be measured in all patients with possible PSC in order to exclude IgG4-related sclerosing cholangitis^(7)^.

Renal involvement occurs in up to one third of patients and is usually multiple and bilateral, presenting as round or wedge-shaped cortical nodules, small peripheral cortical lesions, renal masses (pseudotumors), or renal pelvic involvement. Contrast-enhanced CT typically shows hypodense lesions showing mild contrast enhancement in later phases. On MRI scans, the lesions are isointense/hypointense on T1-weighted sequences and hypointense on T2-weighted sequences^(2,8)^. These imaging findings reflect histopathological features including lymphoplasmacytic infiltration of the renal interstitium, with an increased number of IgG4-positive plasma cells, and fibrosis^(2,8)^. The combined presence of pancreatic and extrapancreatic manifestations further supports the diagnosis of IgG4-related disease, as in the present case.

After clinical improvement with corticosteroid therapy, patients with IgG4-related disease require follow-up, given that there have been reports of an increased relative risk of cancer in the year following diagnosis-including stomach cancer (the most common type of cancer in such patients), lung cancer, prostate cancer, colon cancer, non-Hodgkin lymphoma, bile duct cancer, and thyroid cancer^(9)^. Although the number of studies on this topic is growing, the natural history and long-term prognosis of IgG4-related disease have yet to be well defined. Therefore, patient monitoring and a multisystem evaluation approach, including periodic imaging, are required.

Given that IgG4-related disease can mimic neoplastic lesions, there is a risk that a more aggressive approach might be taken if a diagnosis of IgG4-related disease is not taken into account. The case reported here shows that radiologists should be aware of this possibility in order to establish the correct diagnosis and select the most appropriate therapy.
